# Predictors and neurological consequences of periprocedural cerebrovascular events following transcatheter aortic valve implantation with self-expanding valves

**DOI:** 10.3389/fcvm.2022.951943

**Published:** 2022-10-05

**Authors:** Ferenc Imre Suhai, Andrea Varga, Bálint Szilveszter, Milán Nagy-Vecsey, Astrid Apor, Anikó Ilona Nagy, Márton Kolossváry, Júlia Karády, Andrea Bartykowszki, Levente Molnár, Ádám L. Jermendy, Alexisz Panajotu, Pál Maurovich-Horvat, Béla Merkely

**Affiliations:** ^1^Cardiovascular Imaging Research Group, Heart and Vascular Center, Semmelweis University, Budapest, Hungary; ^2^Department of Medicine, Karolinska Institute, Stockholm, Sweden; ^3^Cardiovascular Imaging Research Center, Massachusetts General Hospital and Harvard Medical School, Boston, MA, United States; ^4^Medical Imaging Center, Semmelweis University, Budapest, Hungary

**Keywords:** cerebral embolism, transcatheter aortic valve implantation, cardiac CT angiography (CTA), stroke, magnetic resonance imaging

## Abstract

**Aims:**

To evaluate the patient- and procedure-related predictors of transcatheter aortic-valve implantation (TAVI)-associated ischemic brain lesions and to assess the effect of silent cerebral ischemic lesions (SCIL) on neurocognitive function.

**Methods and results:**

We investigated 113 consecutive patients with severe aortic stenosis who underwent brain magnetic resonance imaging (MRI) within a week following TAVI. To assess periprocedural cerebral ischemic lesions, diffusion-weighted MRI was utilized. We used multivariate linear regression to identify the independent predictors of TAVI-related ischemic lesion volume (ILV) and periprocedural stroke. Neurocognitive evaluation was performed before and following TAVI at 6-month and one-year follow-up. Following TAVI, a total of 944 new cerebral ischemic lesions were detected in 104 patients (92%). The median ILV was 257 μl (interquartile range [IQR]:97.1–718.8μl) with a median lesion number of 6/patient [IQR:2–10]. The majority of ischemic lesions were clinically silent (95%), while 5% of the lesions induced a stroke, which was confirmed by MRI. Predilatation (β = 1.13[95%CI:0.32–1.93], *p* = 0.01) and the number of valve positioning attempts during implantation (β = 0.28[95%CI:0.06–0.50], *p* = 0.02) increased the log-transformed total ILV. Predilatation (OR = 12.04[95%CI:1.46–99.07], *p* = 0.02) and alternative access routes (OR = 7.84[95%CI:1.01–61.07], *p* = 0.02) were associated with stroke after adjustments for comorbidities and periprocedural factors. The presence of SCILs were not associated with a change in neurocognitive function that remained stable during the one-year follow-up.

**Conclusion:**

While periprocedural ischemic lesions are frequent, most of them are clinically silent and might not impact the patients' neurocognitive function. The number of valve positioning attempts, predilatation, and alternative access routes should be taken into consideration during TAVI to reduce the ILV and risk for stroke.

## Introduction

Aortic stenosis (AS) is the most common valvular disease in developed countries (1, 2). The prevalence is increasing with age, and it has substantial impact on the mortality and morbidity in the elderly population (3). Surgical aortic valve replacement (SAVR) has been the standard treatment for patients with severe AS. Transcatheter aortic valve implantation (TAVI) has emerged as a safe and effective alternative to SAVR in symptomatic patients with high or prohibitive risk and as a valid alternative to AVR in patients with intermediate risk (4–9). TAVI has been expanded to lower risk patient population, according to the 2020 US guideline, and it can be considered for symptomatic patients between the ages of 65 and 80 years and for asymptomatic patients <80 years with an ejection fraction of <50% (10, 11). It has been shown that TAVI is superior to medical therapy and balloon valvuloplasty in patients who are not suitable for open-heart surgery (12, 13) and could potentiate reverse remodeling of the left ventricle (14).

Cerebrovascular events (CVE) after TAVI are among the most worrisome complications, increasing the risk of morbidity and mortality at short- and long-term (15–17). The incidence of CVE after TAVI ranges from 1–11% according to different studies and meta-analyses, and it varies according to the definition, albeit the incidence of periprocedural stroke is slightly lower in patients with new generation devices as compared to patients with first generation valves (17–20). In addition to the clinically apparent ischemic brain lesions, several cerebral magnetic resonance imaging (MRI) studies showed a very high (58–91%) incidence of clinically silent new ischemic lesions after TAVI, regardless of the transcatheter valve type and approach (21–24). Although periprocedural stroke presents only in a small proportion of patients, silent cerebral embolism is a common finding associated with this procedure. Furthermore, the real impact of these silent cerebral ischemic lesions (SCIL) on cognitive function and development of future cerebral complications are still under debate (25). It has been suggested that SCILs after TAVI are associated with an increased risk of dementia, cognitive decline, and depression (26–28).

Our primary aim was to identify patient- and procedure-related predictors of ischemic brain lesions and stroke following TAVI, as well as their occurrence and distribution using diffusion MRI. Our secondary aim was to assess the effect of SCILs on the patients' neurocognitive function.

## Materials and methods

### Study population and design

In a single-center, prospective cohort study, we analyzed consecutive patients who underwent CT angiography (CTA) for pre-TAVI planning and brain MRI following TAVI as part of the RETORIC study (Rule out Transcatheter Aortic Valve Thrombosis with Post Implantation Computed Tomography trial, NCT02826200) (29). The valve implantations were performed between November 2016 and June 2018, and patients were followed up until 1 year.

This study was approved by the local and national ethical committees and was performed in accordance with the Helsinki declaration. Written informed consent was obtained from all patients.

### Image acquisition for TAVI planning

We used the following CTA protocol for every pre-TAVI planning CT: first, we acquired a prospectively ECG triggered non-contrast scan from the entire heart (120 kV, slice thickness of 3 mm, increment 1.5 mm). This was followed by a retrospectively ECG gated CTA of the aorta (from the level of thoracic inlet to the level of the femoral head) and the heart, during a single breath-hold, using a 256-slice CT scanner (Philips Healthcare, 270 ms rotation time, tube voltage of 100–120 kV based on body mass index) for TAVI planning. We administered 75 ml contrast agent with 4.5 ml/s flow, and images were acquired with 1 mm slice thickness and 1 mm increment using iterative reconstruction (iDose^4^ and IMR, Philips Healthcare).

### Cardiac CTA image analysis

**Figure 1:**
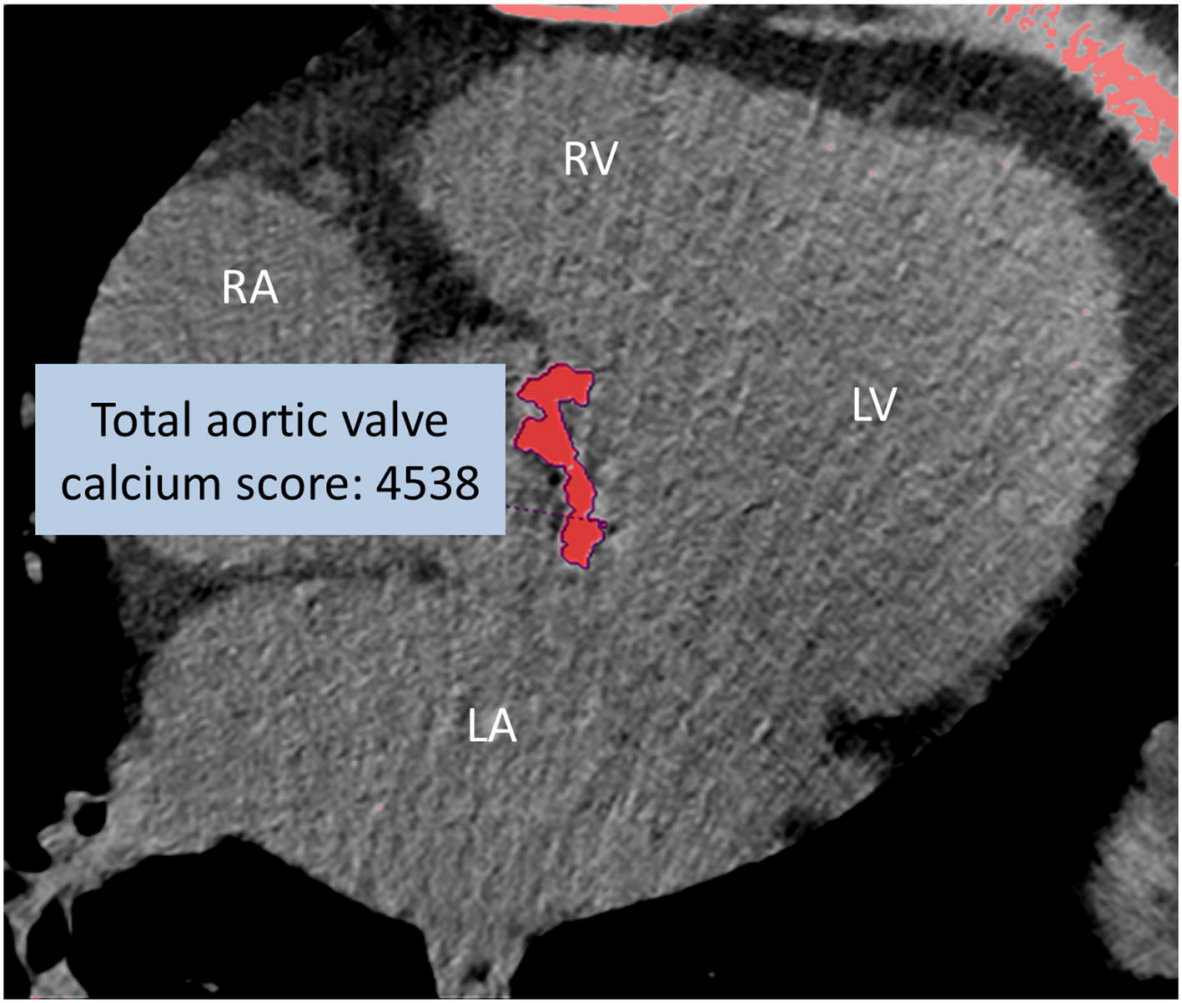
Non-enhanced CT of severe aortic valve calcification (total AVCS: 4538). Calcium scoring of the aortic valve using post-processing software by the Agatston method. RA, right atrium; RV, right ventricle; LA, left atrium; LV, left ventricle.

Two radiologists assessed the calcification of the aortic valve, the annulus, the left ventricular outflow tract, the ascending aorta, and the aortic arch. The severity of calcification was qualitatively graded as mild, moderate, and severe. The aortic valve calcium score (AVCS) was measured on the non-contrast cardiac CT by the Agatston method (Figure 1), with care taken to exclude calcium originating from the extravalvular structures (30), using a semi-automated software tool (Heartbeat-CS, Philips Intellispace v6.0.4). The measurements were performed in a random order, and investigators were blinded to the scan date and patient data.

### TAVI procedure

Prosthetic valves were implanted with the standard technique, by using local anesthesia with conscious sedation during the procedure. Transfemoral route was the preferred access, and the trans-subclavian or transcarotid route was considered an alternative route. Embolic protection devices were not used in this cohort. Only self-expandable valves were used in our study. Adverse events were defined according to the Valve Academic Research Consortium-3 definitions (VARC-3) (31, 32). Procedural factors such as balloon predilation and postdilatation, the number of attempts to position, and events of valve dislocation were evaluated and collected in a dedicated database (Table 1).

**Table 1:** Procedural characteristics.

**Patient data (*N =* 113)**	
Aortic valve calcium score	3,321.6 ± 1,944.7
Bicuspid aortic valve, *n* (%)	15 (13.3)
Access route (TF vs. TS/TC), *n* (%)	105 (92.9) vs. 6 (5.3) vs. 2 (1.8)
Predilatation, *n* (%)	15 (13.3)
CoreValve vs. evolutr vs. portico, *n* (%)	9 (8.0) vs. 75 (66.3) vs. 29 (25.7)
Number of attempts to position	1.7 ± 0.9
Malposition/Migration, *n* (%)	5 (4.4)
Postdilatation, *n* (%)	89 (78.8)
New-onset atrial fibrillation *n* (%)	8 (7.1)
Vascular and acces-related complications, *n* (%)	26 (23.0)
Minor (according to VARC-3 criteria)	17 (15.0)
Major (according to VARC-3 criteria)	9 (8.0)

VARC-3, Valve Academic Research Consortium, TF, Transfemoral, TS, Trans-subclavian, TC, Transcarotid.

Continuous variables are expressed as mean ± standard deviation (SD) and categorical variables are expressed as numbers and percentages.

### Brain MRI examination

**Figure 2:**
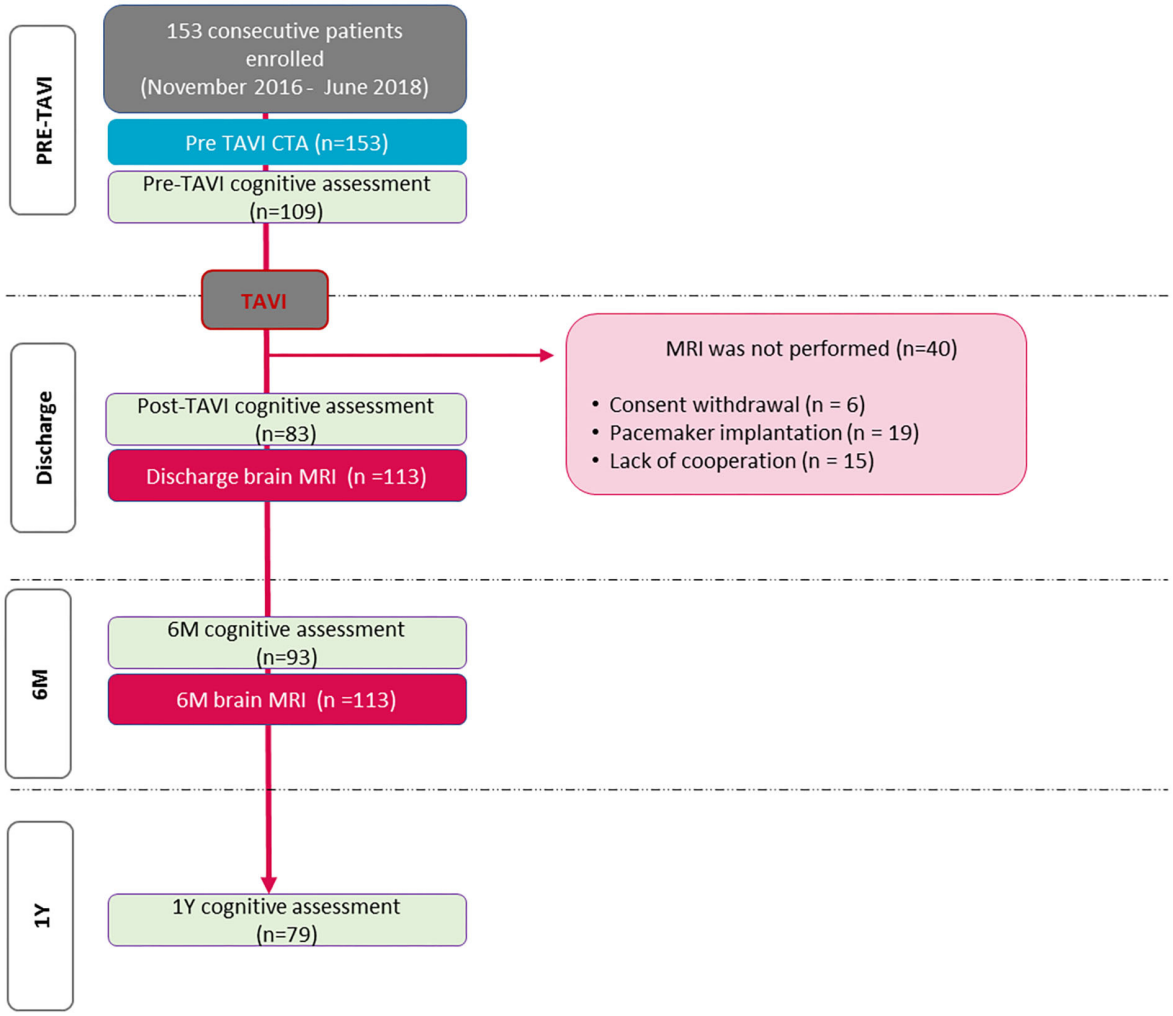
Flowchart of the study.

We performed brain MRI in the first week (4 days after TAVI on average) to detect cerebral ischemic lesions. Patients were excluded, if there was a contraindication to MRI or if they had poor image quality. After applying the abovementioned exclusion criteria, 113 patients were analyzed (Figure 2).

The MRI examinations were performed on a 1.5T MR scanner (Achieva, Philips Medical Systems) using an eight-channel head coil in the first week (mean 4 days) after TAVI (referred to as discharge MRI). Fluid-Attenuated Inversion Recovery (FLAIR), T2-weighted, T2^*^-gradient echo, high resolution 3D T1-weighted gradient echo sequences were obtained with diffusion MRI. MRI was repeated at 6-month follow-up (6M) in order to assess the gliotic transformation of procedural ischemic lesions.

Diffusion MRI acquisitions were performed using a single shot spin echo, echo-planar imaging sequence in 32 diffusion encoding directions with b = 800 s/mm^2^ and one b = 0 measurement. Whole brain coverage was obtained with 2 mm-thick contiguous axial slices. From the diffusion, MRI dataset averaged diffusion-weighted images commonly referred to as “trace”, and mean diffusivity and ADC maps were automatically derived and used to calculate the ischemic lesion volume (ILV). New ischemic lesions were detected at postprocedural imaging on diffusion-weighted imaging (DWI), and they were considered completely resolved if neither DWI nor FLAIR positive lesions were detected in the same location at follow-up; gliotic transformation was considered if there was FLAIR hyperintensity in the same location of the discharge DWI positive lesion.

### Ischemic lesion volume measurement

The number, localization, and three perpendicular diameters of all lesions with restricted diffusion images were recorded using an AGFA PACS workstation (Impax 6.5.2.657, Agfa HealthCare). ILV was calculated as the sum of lesion volumes using the formula of a x b x c x 0.52 (a, b, and c are the three lesion diameters) (33). The ILV measurements were performed in a random order and the investigator was blinded to the scan date and patient data.

### Neurocognitive assessment

Patients underwent a serial evaluation of the cognitive status, pre-TAVI, and post-TAVI before hospital discharge, 6-month follow-up (6M), and 1-year follow-up (1Y) following TAVI. We used the Hungarian version of the Addenbrooke's Cognitive Assessment (ACE) test (34), which incorporated the Mini-Mental State Examination (MMSE), and the evaluation was performed by one of the two trained investigators blinded to CTA and MRI data. Among all enrolled patients, 113 participants completed the pre-TAVI, 83 subjects completed the post-TAVI, 93 subjects completed the 6M, finally 79 patients completed the 1Y cognitive tests. Patients with periprocedural stroke (6/113, 5.3%) were excluded from the further neurocognitive assessment.

### Statistical analysis

Continuous variables are presented as mean and standard deviation, whereas categorical variables are presented as frequency with percentages. Categorical variables were compared using the chi-squared test. The Kruskal-Wallis test was used to analyze the association between ILV and the number of positioning of the valve during TAVI. Because of non-normal distribution of ILV, data were log-transformed. The univariate linear regression analysis was performed to detect the association between patient- and procedure-related risk factors and log-transformed ILV. The multivariate linear regression models were performed using the backward method.

We also aimed to identify predictors of periprocedural stroke using univariate and multivariate logistic regression. Repeated-measures analysis of variance was performed to evaluate changes in neurocognition over time; pairwise differences were assessed using Duncan's multiple comparison test. A *p*-value < 0.05 was considered statistically significant. All calculations were performed using SPSS software (SPSS version 23; IBM Corp.).

## Results

In total, 113 patients were included in the analysis (mean age: 79.2 ± 6.7 years, 44.2% women, and mean BMI: 27.3 ± 4.7 kg/m^2^). Overall, 23.9% (27/113) of the patients had prior myocardial infarction, 90.3% (102/113) had hypertension, and 65.5% (74/113) had hyperlipidaemia. Oral anticoagulant medication was administered in 29.2% (33/113), while 74.3% (84/113) of the patients received antiplatelet therapy. Patient characteristics and imaging parameters are summarized in Table 2.

**Table 2:** Demographic parameters and cardiovascular risk factors.

**Patient data (*N =* 113)**	
Age (years)	79.2 ± 6.7
Female sex, *n* (%)	50 (44.2)
BMI (kg/m^2^)	27.3 ± 4.7
Diabetes, *n* (%)	54 (47.8)
Hypertension, *n* (%)	102 (90.3)
Hyperlipidemia, *n* (%)	74 (65.5)
Previous AMI, *n* (%)	27 (23.9)
PAD, *n* (%)	57 (50.4)
Atrial fibrillation, *n* (%)	38 (33.6)
Previous TIA/stroke, *n* (%)	15 (13.3)
Chronic kidney disease	64 (56.6)
Antiplatelets, *n* (%)	84 (74.3)
Anticoagulants, *n* (%)	33 (29.2)

BMI, Body mass index; AMI, Acute myocardial infarction; PAD, Peripheral artery disease; TIA, Transient ischemic attack.

Continuous variables are expressed as mean ± standard deviation (SD) and categorical variables are expressed as numbers and percentages.

### Procedural characteristics

Procedural characteristics and procedural complications are summarized in Table 1. Prosthetic valves were implanted successfully in all patients (Medtronic CoreValve 8.0%, Medtronic CoreValve Evolut R 66.3%, Portico 25.7%). The mean AVCS was 3,332 ± 1,944, and 13.3% of the patients had a bicuspid aortic valve (BAV). The transfemoral approach was used in 105 patients (92.9%), the trans-subclavian access in six cases (5.3%), and the transcarotid route in two patients (1.8%). Balloon predilatation was performed in 15 patients (15.3%), while most of the valves (78.8%) were postdilated. Predilatation was performed in the case of the heavily calcified native aortic valve, according to the operators' visual judgment; however, no significant difference in AVCS could be observed in patients with predilatation compared to those without predilatation (median AVCS: 2,774 [IQR:1,885–4,271] vs. median AVCS: 3,612 [IQR:1,847.4–6,366]; *p* = 0.44). The mean number of positional attempts was 1.7 ± 0.9. In 60 (53.1%) cases, the implantation was successful at the first positional attempt, in 39 (34.5%) cases at the second, and in 14 patients (12.4%) at the third or fourth time. According to the VARC-3 criteria, nine patients had major and 17 patients had minor vascular and access-related complications.

### Cerebral embolization after TAVI

**Figure 3:**
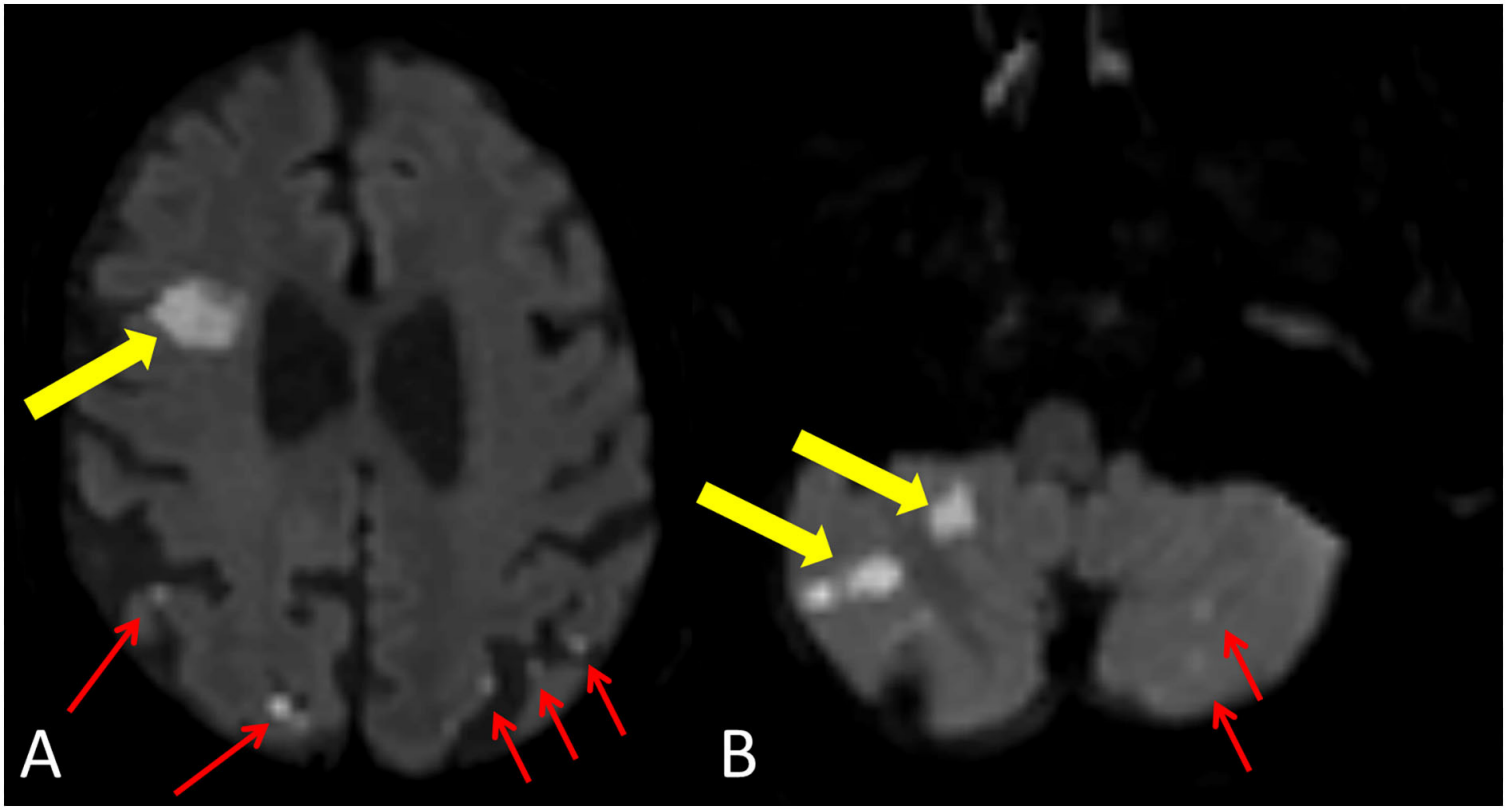
New ischemic lesion after TAVI. Yellow arrows demonstrate a larger lesion with restricted diffusion in the right frontal lobe **(A)** and in the right cerebellar hemisphere **(B)**. Red arrows show smaller cortical-subcortical lesions with restricted diffusion in the left and right parietal lobes **(A)** and in the left cerebellar hemisphere **(B)**.

A total of 104 patients (92.0%) had new cerebral ischemic lesions on discharge MRI (Figure 3), among them six patients had periprocedural stroke. The median number of lesions per patient was six (IQR: 2–10), and the median ILV was 257.3 μl (IQR: 97.1–718.8 μl). In addition, 944 new ischemic brain lesions were found on brain MRI, most of the lesions were supratentorial (781/944, 81.9%), and the majority were located in the cortical–subcortical area (796/944, 84.3%). The left and right cerebral and cerebellar hemispheres were equally affected (Table 3). On the 6M MRI, 46/113 (40.7%) patients had gliotic transformation on FLAIR images.

**Table 3:** Results of postprocedural assessment with MRI.

**Patient data (*N =* 113)**	
Patients with new cerebral ischemic lesions, *n* (%)	104 (92.0)
Patients with periprocedural stroke, *n* (%)	6 (5.3)
Number of lesions per patient	6 (2–10)
Ischemic load per patient (μl)	257.3 [97.1–718.8]
Number of lesions: left vs. right, *n* (%)	500 (52.97) vs. 444 (47.03)
Volume of lesions: left vs. right (μl)	123.3 [29.7–357.9] vs. 89.1 [14.6–226.1]
Number of lesions: supra- vs. infratentorial, *n* (%)	781 (82.7) vs. 163 (17.3)
Volume of lesions: supra- vs. infratentorial (μl)	58.3 [14.58–215.6] vs. 0.0 [0.0–53.1]
Cortical-subcortical lesions, *n* (%)	796 (83.4)
Deep lesions, *n* (%)	158 (16.6)
Lesions < 5 mm, *n* (%)	558 (59.1)
Lesions 5–10 mm, *n* (%)	332 (35.2)
Lesions > 10 mm, *n* (%)	54 (5.7)

Continuous variables are expressed as median and interquartile ranges [IQR] and categorical variables are expressed as numbers and percentages.

### Predictors of ischemic lesion volume and stroke after TAVI

**Figure 4:**
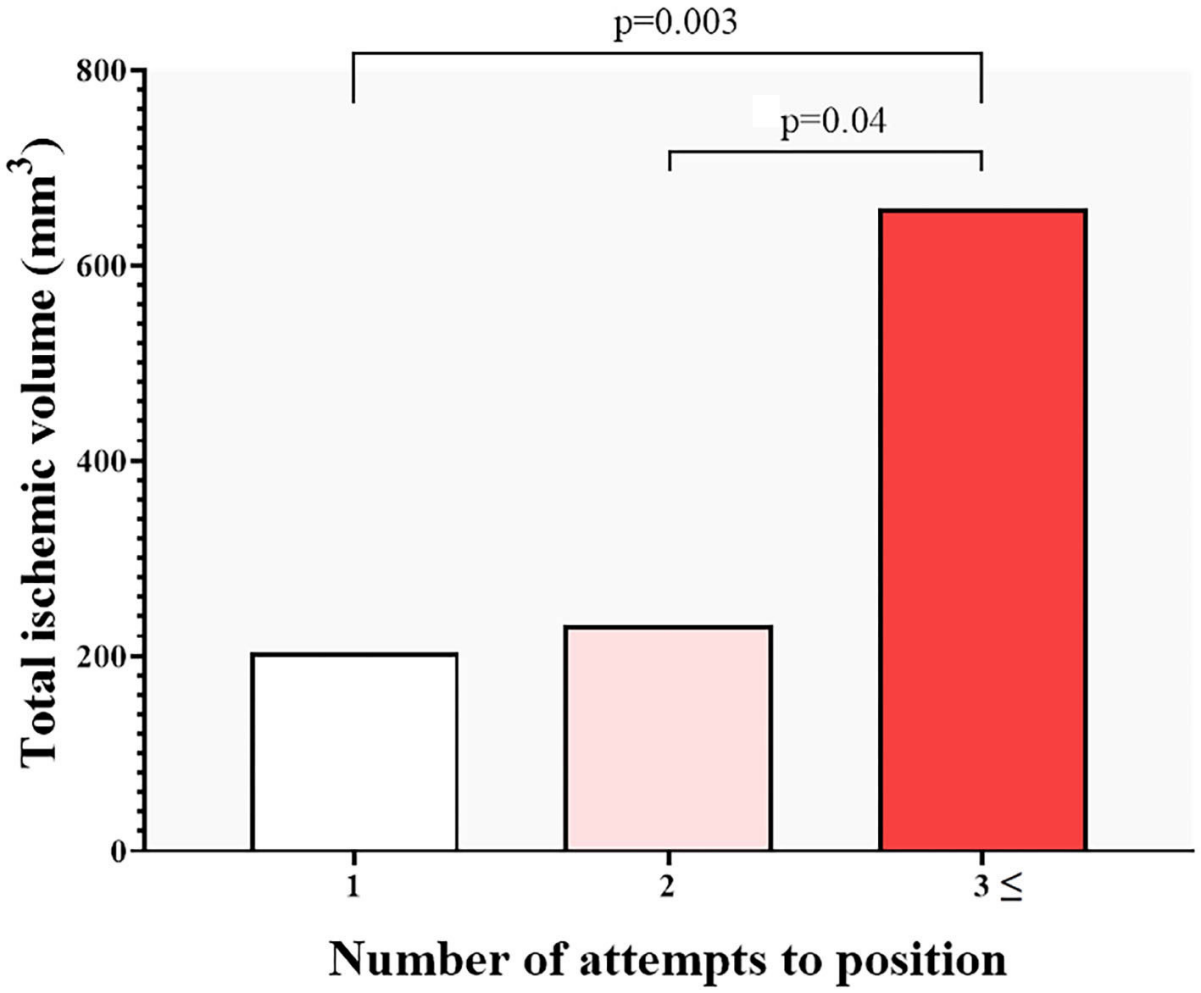
Total ischemic volume on MRI and the number of TAVI positioning attempts. The number of procedural manipulations shows a strong correlation with the ischemic lesion volume (ILV), Three or more positioning attempts of the device resulted in significantly increased ILV.

We evaluated clinical and imaging parameters for association with ILV and stroke. Age, cardiovascular risk factors, aortic calcification, access route, valve type and size, and postdilatation did not show any association with ILV (all non-significant see, *p* > 0.05 Table 4). On univariate analysis, sex, AVCS, number of valve positioning attempts, and predilatation showed an association with log-transformed ILV. AVCS was not an independent predictor of log-transformed ILV after adjustments. Regarding ILV, it seems that the manipulations during TAVI are more relevant than the AVCS: positioning the device three or more times resulted in a significant increase in ILV (Figure 4). On multivariate linear regression analysis, predilatation (β = 1.13, 95% CI:0.32–1.93; *p* = 0.01), and positioning attempts (β = 0.28, 95 % CI: 0.06–0.50; *p* = 0.02) were independent predictors of log-transformed ILV after adjusting for covariates using the backward method (Table 4).

**Table 4:** Multivariate linear regression analysis of the predictors of total ischemic volume.

	**Univariate**				**Multivariate**			
	**β**	**95% CI, lower-upper**		***p***	**β**	**95% CI, lower-upper**		***p***
Sex	***0.48***	***0.10***	***0.86***	***0.02***	0.25	−0.15	0.66	0.22
New-onset atrial fibrillation	0.65	−0.11	1.40	0.09				
Previous AF	0.39	−0.02	0.80	0.06	0.33	−0.04	0.71	0.08
Anticoagulant therapy	0.002	−0.008	0.01	0.65				
Previous stroke/TIA	0.14	−0.45	0.74	0.64				
Aortic valve ca score	***0.00***	***0.00***	***0.00***	***0.02***	0.00	0.00	0.00	0.055
Bicuspid aortic valve	−0.22	−1.03	0.59	0.59				
Alternative access route	0.50	−0.26	1.26	0.19	0.68	−0.04	1.40	0.06
**Predilatation**	***0.93***	***0.08***	***1.79***	***0.03***	***1.13***	***0.32***	***1.93***	***0.01***
Malposition	0.24	−0.71	1.19	0.62				
Postdilatation	−0.17	−0.65	0.31	0.49				
**Number of attempts to position**	**0.23**	**0.03**	**0.44**	**0.03**	***0.28***	***0.06***	***0.50***	***0.02***

AF, Atrial fibrillation; CI, Confidence interval; TIA, Transient ischemic attack. Numbers marked in bold are significant predictors of the outcome based on multivariate analysis (p < 0.05).

On multivariate logistic regression analysis, we found that predilatation (OR:12.04; 95%CI: 1.46–99.07; *p* = 0.02) and alternative access route (OR: 7.84; 95%CI: 1.01–61.07; *p* = 0.049) were independent predictors of periprocedural stroke (Table 5).

**Table 5:** Multivariate logistic regression analysis of the predictors of periprocedural stroke.

	**Univariate**			**Multivariate**		
	**OR**	**95% CI, lower-upper**	***p***	**OR**	**95% CI, lower-upper**	***p***
Sex	2.65	0.47–15.11	0.27			
New-onset atrial fibrillation	2.86	0.29–27.92	0.37			
Previous AF	−0.99	0.17–5.64	0.99			
Anticoagulant therapy	−0.04	−0.75–1.23	0.77			
PAD	0.98	0.19–5.08	0.98			
Previous stroke/TIA	1.58	0.17–14.72	0.69			
Aortic valve ca score	1.00	1.00–1.00	0.99			
Bicuspid aortic valve	3.62	0.60–21.74	0.21			
**Alternative access route**	***8.42***	***1.28***–***55.53***	***0.03***	***7.84***	***1.01***–***61.07***	***0.049***
**Predilatation**	***12.88***	***1.80***–***92.27***	***0.01***	***12.04***	***1.46***–***99.07***	***0.02***
Malposition	0.00	0.00–0.00	1.00			
Postdilatation	0.52	0.09–3.01	0.46			
Number of attempts to position	1.49	0.81–2.75	0.20			

AF: Atrial fibrillation; CI: Confidence interval; PAD: Peripherial artery disease; TIA: Transient ischemic attack Numbers marked in bold are significant predictors of the outcome based on multivariate analysis (p < 0.05).

### Neurocognitive function

**Figure 5:**
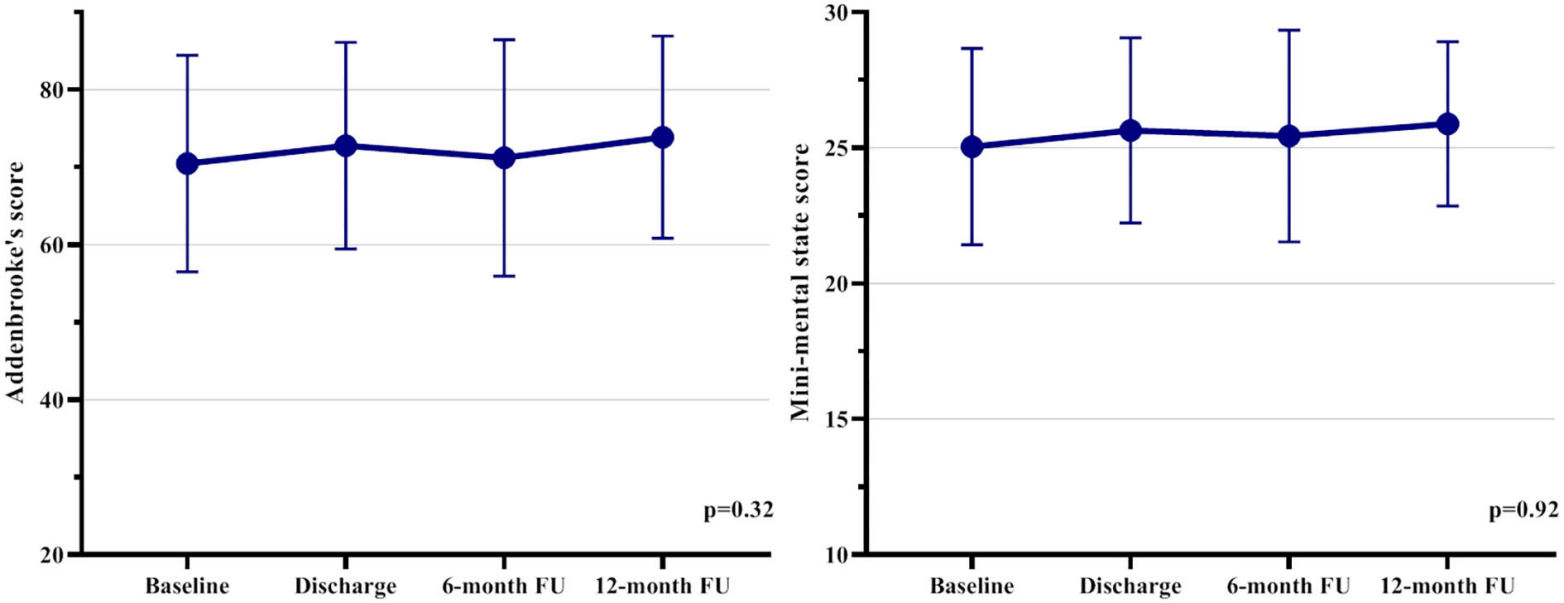
Neurocognitive examination results in the 79 patients based on serial assessments. The overall neurocognitive function was stable during the one-year follow-up.

Among all patients, 79 out of 113 patients had a serial neurocognitive assessment and post-TAVI MRI, and these subjects were included in our subanalysis. The overall cognitive performance of the cohort was stable over the 1Y follow-up period (Figure 5), with mean baseline, discharge, 6M Addenbrooke's score, and 1Y Addenbrooke's score of 72.3 ± 13.1, 74.8 ± 14.2, 72.8 ± 16.6, and 73.4 ± 13.4 (*p* = 0.32) and an MMS score of 25.9 ± 2.8, 26.1 ± 3.5, 25.8 ± 4.1, and 26.3 ± 3.0, *p* = 0.92, respectively (Table 6). We found that neither ILV nor the presence of gliotic transformation of these procedural lesions was associated with neurocognitive change at any time during the follow-up period (at discharge, at 6M, at 1Y, *p* > 0.05 for all).

**Table 6:** Results of serial neurocognitive assessments.

	**Baseline**	**Discharge**	**6-month follow-up**	**12-month follow-up**	***p***
Mini-mental state score	25.9 ± 2.8	26.1 ± 3.5	25.8 ± 4.1	26.3 ± 3.0	0.92
Adenbrook's score	72.3 ± 13.1	74.8 ± 14.2	72.8 ± 16.6	73.4 ± 13.4	0.32

Parameters are shown as mean ± SD.

## Discussion

The main findings of our study are as follows: (1) we found that 92% of the patients had new cerebral ischemic lesions; however, most of them were clinically silent; (2) balloon predilatation and the number of valve positioning attempts during the procedure were independently associated with a larger log-transformed ILV, whereas predilatation and alternative access route were associated with periprocedural stroke; and (3) the ILV was not associated with cognitive decline after TAVI.

Despite the extensive literature on CVE and SCIL risk factors during TAVI, the identified predictors differ from study to study, highlighting the great complexity of patient- and procedure-related factors (15, 17, 19–23, 28, 35–45). Although CVE is relatively rare, it is the most worrisome complication in this frail patient population with multiple comorbidities, which is linked to poor outcomes. Nombela-Franco et al. found that balloon postdilatation and valve dislodgement/embolization were predictors of acute CVE, and new-onset atrial fibrillation was a predictor of subacute CVE (15). Keiko et al. found that self-expandable valves were associated with an increased risk of acute cerebral embolization on MRI (39). A meta-analysis showed that female sex, chronic kidney disease, level of experience, and new-onset atrial fibrillation were predictors of CVE post-TAVI (19). Regarding the access site, Rodés et al. found no difference when comparing transfemoral vs. transapical approaches (23); however, Eggebrecht et al. (16) found an association between stroke and the type of approach, with transapical TAVI carrying the lowest risk of stroke. A meta-analysis from Lu et al. found that transcarotid access was associated with an increased risk of 30-day mortality and with an increased risk of 30-day neurovascular complications (46). A nationwide study from Sweden found that reduced renal function, diabetes, history of stroke, age, and male sex were risk factors for developing stroke after TAVI (47). Also, a recent meta-analysis showed that next-generation devices can decrease TAVI-related complications, including periprocedural stroke (18). We identified predilatation and valve positioning maneuver as important predictors of larger ILV, whereas predilatation and access route were risk factors of periprocedural stroke.

SCILs are more frequent after TAVI, but their impact on neurocognitive function still remains controversial (24, 27, 28, 36, 37). Various cerebral MRI studies showed a very high (58–91 %) incidence of new ischemic lesions after TAVI, regardless of the transcatheter valve type and approach (22–24, 38). Several different predictors for SCIL have been identified: Carlo et al. showed that baseline age-related white matter damage was an independent predictor of the occurrence of SCILs together with the use of non-balloon-expandable prostheses (36). A recent meta-analysis showed that diabetes, kidney disease, and predilatation increased the overall risk for SCIL (28).

We found that the number of positioning maneuvers of the device resulted in a significantly increased log-transformed ILV. However, AVCS did not show a correlation with ILV. Importantly, the transcatheter valve type, access route, or the presence of BAV did not influence the log-transformed ILV either. Although alternative access route did not appear to be a significant predictor of ILV on multivariate analysis, an increasing tendency in ILV could be observed and the lack of statistical significance regarding the association between ILV and alternative access route could be explained by the relatively low number of alternative access. Notably, some studies found an association between AVCS and cerebral embolization, as well as acute periprocedural CVE (48, 49). According to our study, it appears that aortic valve calcification has limited associations with CVE.

In a recent study, Fan et al. published that patients with BAV had more cerebral ischemic lesions following TAVI (50). In our study, we found that AVCS was higher in patients with BAV compared to patients with tricuspid valves; however, the procedural characteristics and ILV did not differ between the two groups (Table 7).

**Table 7:** Procedural characteristics of patients with the bicuspid and tricuspid valves.

**Patient data**	**Bicuspid**	**Tricuspid**	***p***
(*n* = 113)	(*n* = 15)	(*n* = 98)	
Aortic valve calcium score	***4,913** **±2,800***	***3,078** **±1,668***	*** < 0.001***
Ischemic load (mm^3^)	4,789 ± 2,1800	4,086 ± 1,7450	0.95
Access route (TF vs. TS vs. TC), *n* (%)	***12 (80.0) vs. 3 (20.0)***	***93 (94.9) vs. 5 (5.1)***	***0.04***
Predilatation, *n* (%)	3 (20.0)	12 (12.2)	0.41
CoreValve vs. portico, *n* (%)	13 (86.7) vs. 2 (13.3)	71 (72.4) vs. 27 (27.6)	0.24
Malposition/migration, *n* (%)	0 (0.0)	3 (3.1)	1.00
Postdilatation, *n* (%)	14 (93.3)	75 (76.5)	0.14
Stroke, *n* (%)	2 (13.3)	4 (4.1)	0.14
Vascular and acces-related complications *n* (%)			
Minor (according to VARC-3 criteria)	2 (13.3)	15 (15.3)	0.84
Major (according to VARC-3 criteria)	2 (13.3)	8 (8.2)	0.51

VARC-3, Valve Academic Research Consortium; TF, Transfemoral; TS, Transsubclavian; T, Transcarotid.

Continuous variables are expressed as mean ± standard deviation (SD) and categorical variables are expressed as numbers and percentages.

The bold values indicate the significant differences between the groups (*p* < 0.05).

The results of our study showed that 5.3% of the patients had periprocedural stroke, which is concordant with the findings of Auffret and colleagues (19). Based on our results predilatation and alternative access route were associated with periprocedural stroke. Predilatation was usually performed if there was heavy leaflet calcification by the visual estimation of the interventional cardiologist, but AVCS did not differ between patients with or without predilatation. The association between the number of device positioning maneuvers and stroke could not be observed; however, the stroke incidence was low.

Some studies revealed a neurocognitive decline after TAVI (26, 28); however, Kahlert et al. found no significant changes in cognitive function (38). A subgroup analysis from a recent meta-analysis showed that, despite new cerebral lesions following TAVI, there is a cognitive improvement in 19% and impairment in only 7% (37) of the subjects. They found that using a cerebral embolic protection device was associated with a decreased prevalence of cognitive decline up to 1-week post-TAVI, and pre-TAVI cognitive impairment had an association with post-TAVI cognitive improvement at 6-month. It has to be acknowledged that studies with longer follow-up [i.e., Vermeer et al. with 3.6 years follow-up (26)] might better identify an association with cognitive dysfunction compared to studies with a shorter follow-up (28). In our study, the neurocognitive function was stable during the 1Y period, and we could not find any association between ILV or gliotic transformation of the procedural lesions and changes in neurocognitive function. To our knowledge, this is the largest patient population who underwent brain MRI and had a one-year-long serial neurocognitive assessment after TAVI, and this study is the first to report an association between the number of device positioning maneuvers and ILV.

Procedural complications such as CVE and SCILs still remain a problem, and the effect of SCIL on neurocognitive function is controversial; therefore, identifying the patient- and procedure-related risk factors for CVE and SCIL are crucial to achieve the best long-term outcome.

## Limitations

Some limitations of the present study must be acknowledged. Our single-center study enrolled 153 patients for the current evaluation, but we included 113 patients with brain MRI. Patients who received a pacemaker post-TAVI or could not cooperate with the brain MRI were excluded, which might have led to selection bias. This together with a proportion of patients who did not participate in the serial neurocognitive assessment could influence neurocognitive decline rates. Also, longer follow-up could better find the association between SCIL and neurocoginitive decline. Alternative access route and predilatation was used in a limited number of patients that could possibly limit the generalizability of our findings.

## Conclusion

In the present study, we found that more procedural manipulations and predilatation resulted in larger log-transformed ILV on discharge MRI following TAVI. We identified a new procedural risk factor, namely, the number of positioning maneuvres of the valve that should be taken into consideration during TAVI. However, the clinically silent lesions did not influence the patient's neurocognitive function during 1Y. Predilatation and alternative access route were associated with stroke after TAVI in our study.

## Data availability statement

The raw data supporting the conclusions of this article will be made available by the authors, without undue reservation.

## Ethics statement

The studies involving human participants were reviewed and approved by National Institute of Pharmacy and Nutrition. The patients/participants provided their written informed consent to participate in this study.

## Author contributions

Conceptualization: FS, BS, PM-H, AA, and AN. Methodology: FS and BS. Software: FS and MN-V. Validation: FS, MN-V, AP, BS, and ÁJ. Formal analysis: BS, MN-V, and MK. Investigation and writing-original draft preparation: FS. Resources: LM and AP. Data curation: JK and AB. Writing-review and editing: AA, AN, JK, AB, ÁJ, MK, and AV. Visualization: FS and MN-V. Supervision: PM-H and BM. Project administration: AN, PM, and BM. Funding acquisition: BM and PM-H. All authors contributed to the article and approved the submitted version.

## Funding

This study was supported by the National Research, Development and Innovation Office of Hungary (NKFIA; Project No. NVKP_16-1–2016-0017-‘National Heart Program') and by the Thematic Excellence Programme (2020-4.1.1.-TKP2020) of the Ministry for Innovation and Technology in Hungary, within the framework of the Therapeutic Development and Bioimaging thematic programmes of the Semmelweis University. AN was supported by the János Bolyai Scholarship of the Hungarian Academy of Sciences.

## Conflict of interest

The authors declare that the research was conducted in the absence of any commercial or financial relationships that could be construed as a potential conflict of interest.

## Publisher's note

All claims expressed in this article are solely those of the authors and do not necessarily represent those of their affiliated organizations, or those of the publisher, the editors and the reviewers. Any product that may be evaluated in this article, or claim that may be made by its manufacturer, is not guaranteed or endorsed by the publisher.

## Abbreviations

ACE: Addenbrooke's cognitive assessment
AS: aortic stenosis
AVCS: aortic valve calcium scor
CTA: computed tomography angiography
DWI: diffusion-weighted imaging
ILV: ischemic lesion volume
FLAIR: fluid attenuated inversion recovery
MRI: magnetic resonance imaging
MMSE: mini-mental state examination
SAVR, surgical aortic valve replacement, SCIL, silent cerebral ischemic lesion, TAVI: transcatheter aortic valve implantation
6M: 6-month follow-up
1Y: one-year follow-up.
